# High uptake of antiretroviral therapy among HIV-positive TB patients receiving co-located services in Swaziland

**DOI:** 10.1371/journal.pone.0196831

**Published:** 2018-05-16

**Authors:** Ishani Pathmanathan, Munyaradzi Pasipamire, Sherri Pals, E. Kainne Dokubo, Peter Preko, Trong Ao, Sikhathele Mazibuko, Janet Ongole, Themba Dhlamini, Samson Haumba

**Affiliations:** 1 Division of Global HIV and TB, United States Centers for Disease Control and Prevention, Atlanta, Georgia, United States of America; 2 Epidemic Intelligence Service, United States Centers for Disease Control and Prevention, Atlanta, Georgia, United States of America; 3 Swaziland National AIDS Programme, Ministry of Health, Mbabane, Swaziland; 4 Division of Global HIV and TB, United States Centers for Disease Control and Prevention, Ezulwini, Swaziland; 5 University Research Co., LLC, Mbabane, Swaziland; 6 Swaziland National TB Control Program, Ministry of Health, Manzini, Swaziland; IAVI, UNITED STATES

## Abstract

**Background:**

Swaziland has the highest adult HIV prevalence and second highest rate of TB/HIV coinfection globally. Recently, the Ministry of Health and partners have increased integration and co-location of TB/HIV services, but the timing of antiretroviral therapy (ART) relative to TB treatment—a marker of program quality and predictor of outcomes—is unknown.

**Methods:**

We conducted a retrospective analysis of programmatic data from 11 purposefully-sampled facilities to evaluate timely ART provision for HIV-positive TB patients enrolled on TB treatment between July-November 2014. Timely ART was defined as within two weeks of TB treatment initiation for patients with CD4<50/μL or missing, and within eight weeks otherwise. Descriptive statistics were estimated and logistic regression used to assess factors independently associated with timely ART.

**Results:**

Of 466 HIV-positive TB patients, 51.5% were male, median age was 35 (interquartile range [IQR]: 29–42), and median CD4 was 137/μL (IQR: 58–268). 189 (40.6%) were on ART prior to, and five (1.8%) did not receive ART within six months of TB treatment initiation. Median time to ART after TB treatment initiation was 15 days (IQR: 14–28). Almost 90% started ART within eight weeks, and 45.5% of those with CD4<50/μL started within two weeks. Using thresholds for “timely ART” according to baseline CD4 count, 73.3% of patients overall received timely ART after TB treatment initiation. Patients with CD4 50-200/μL or ≥200/μL had significantly higher odds of timely ART than patients with CD4<50/μL, with adjusted odds ratios of 11.5 (95% confidence interval [CI]: 5.0–26.6) and 9.6 (95% CI: 4.6–19.9), respectively. TB cure or treatment completion was achieved by 71.1% of patients at six months, but this was not associated with timely ART.

**Conclusions:**

This study demonstrates the relative success of integrated and co-located TB/HIV services in Swaziland, and shows that timely ART uptake for HIV-positive TB patients *can* be achieved in resource-limited, but integrated settings. Gaps remain in getting patients with CD4<50/μL to receive ART within the recommended two weeks post TB treatment initiation.

## Introduction

In 2014, tuberculosis (TB) surpassed HIV as the leading cause of death from an infectious disease worldwide and, despite advances in prevention and treatment, it remains the leading cause of morbidity and mortality among persons living with HIV (PLHIV) [1]. In Swaziland, the burden of both diseases is particularly high. In 2016 it had the highest HIV prevalence in the world (27% for adults aged 15–49), a TB incidence rate of 398 cases per 100,000 population, and the second highest prevalence of TB and HIV coinfection (with 70% of TB patients estimated to be HIV-positive) [2–4].

To reduce the burden of TB among PLHIV, the World Health Organization (WHO) has long recommended a set of collaborative TB/HIV activities including TB preventive therapy, intensified TB case finding, and TB infection control measures for PLHIV [5]. Additionally, TB patients should be screened for undiagnosed HIV infection and initiated on early antiretroviral therapy (ART) if found to be HIV-positive, which has been shown in randomized controlled trials to reduce mortality as well as loss to follow-up [5–9]. Current guidelines recommend that ART is started within two weeks of TB treatment initiation for patients with profound immunosuppression (a CD4 count less than 50 cells/μL) to reduce morbidity and mortality, and within eight weeks for all other cases (with the caution that immediate ART in the context of TB meningitis may worsen outcomes) [5,10–12].

In accordance with WHO guidelines, the Swaziland Ministry of Health and partners have been implementing collaborative TB/HIV activities over recent years, with most public facilities now providing co-located TB and HIV services. As of 2014, 97% of TB patients had a known HIV status. However, only 79% of notified HIV-positive TB patients were reported by WHO to have initiated ART, and TB-related mortality remained unacceptably high among PLHIV (135 deaths per 100,000, compared with 51 deaths per 100,000 within the general population), even in the face of declining TB notification rates [1, 13]. In addition, the overall timing of ART initiation relative to TB treatment in Swaziland—a marker of TB/HIV program quality and predictor of outcomes—remained largely unknown.

We conducted a retrospective review of routine programmatic facility data to evaluate the provision of timely ART for HIV-positive TB patients in Swaziland, in order to inform recommendations to the National TB Control and National AIDS programs for improving collaborative TB/HIV services.

## Methods

### Study design and setting

This study was a retrospective cohort review of programmatic data from eleven TB and HIV care and co-located treatment sites, purposefully selected to ensure representation of all four regions in Swaziland and all public facility types (four hospitals, four health centers, and three clinics). Criteria for site selection included providing both TB and HIV services, and being operational for at least one year prior to study initiation. As of quarter two of fiscal year 2014, there were 649 TB patients seen in the study sites, of whom 427 (66%) were HIV-positive.

The integrated approach to TB and HIV services in place in these facilities by the time of this study was characterized by ART clinics offering TB screening, diagnosis and isoniazid preventive therapy in addition to routine HIV services, and their co-located TB clinics offering HIV testing, cotrimoxazole preventive therapy, and ART initiation and follow-up (including defaulter tracing) in addition to routine TB services. All patients co-infected with TB and HIV received treatment for both diseases at TB clinics (where their HIV chronic care records were temporarily located), and long term ART was subsequently continued at ART clinics following completion of TB treatment. Linkage of patients between ART and TB clinics was coordinated (especially at large facilities) by expert clients and nursing support. Success of this model was additionally facilitated through creation of national and regional TB/HIV coordinating committees and champions beginning in 2007, declaration of TB as an emergency for PLHIV, decentralization of treatment services to primary health clinics, task-shifting of ART initiation to TB clinic nurses, and social mobilization and educational campaigns to garner community and healthcare worker support.

### Participant sampling and data collection

We assessed ART uptake among a cohort of HIV-positive TB patients. TB facility registers were examined to identify a cohort of eligible patients 15 years and older, who were enrolled on TB treatment between July 1 to November 30, 2014, and had an unknown or positive HIV status. Patients with initially unknown HIV status who were not subsequently found within HIV care and treatment facilities or the Swaziland electronic ART patient monitoring record system were assumed not to have been diagnosed with HIV, and excluded (n = 7). Using standardized data collection forms, anonymous individual-level data were abstracted from facility TB and multidrug-resistant TB registers, pre-ART and ART registers, TB treatment cards, and HIV care booklets. Supplemental data were collected from laboratory and/or pharmacy records as needed. Information abstracted included patient demographics, TB and HIV treatment eligibility, treatment regimens and timing, and clinical and treatment outcomes.

### Data analysis

We defined timely ART per WHO guidelines as ART started within two weeks of TB treatment initiation for patients with profound immunosuppression (CD4 count <50 cells/μL), and within eight weeks for all other cases. For the purposes of this analysis, we also considered ART initiation to have been timely for patients with a missing CD4 cell count only if it was started within two weeks, because we assume that in the absence of a CD4 test the patient should be treated more conservatively.

Descriptive statistics, including frequencies, means and medians were estimated using STATA^®^ software (StataCorp. 2013. *Stata Statistical Software*: *Release 13*. College Station, TX: StataCorp LP), version 13.0. The primary outcome of interest was timing of ART relative to TB treatment, with TB treatment outcome for co-infected patients as a secondary outcome of interest. Logistic regression models, adjusted for within-clinic correlation using empirical sandwich standard errors, were used to estimate odds ratios and 95% confidence limits for variables of interest (including TB treatment outcome). We then fit a multiple regression model including all variables with p<0.20 in a univariate model and report adjusted odds ratios and 95% confidence intervals (CIs) from that model. A p-value of <0.05 was considered statistically significant in the multiple regression model.

### Ethical statement

This study received ethical approval by the Institutional Review Board at the United States Centers for Disease Control and Prevention (CDC), and by the Swaziland Scientific and Ethics Committee. Informed consent was waived for patient record abstraction that was considered routine, anonymous, and of minimal risk to subjects.

## Results

### Patient characteristics

Data were abstracted and analyzed for 466 HIV-positive TB patients, of whom 240 (51.5%) were male. Median age was 36 years (interquartile range [IQR]: 31–43) for men, and 32 (IQR: 27–40) for women. About one third (34.8%) received their care at a clinic, 24.0% at a health center, and 41.2% at a hospital. Eighty-four percent (83.9%) of TB cases were documented as pulmonary rather than extrapulmonary. The majority of patients were new cases (84.3%), followed by relapsed cases (8.4%). Sputum was collected from 77.5% of patients, and *Mycobacterium tuberculosis* was detected by GeneXpert MTB/RIF^®^ in 69.8% of these cases. Median CD4 cell count at the start of TB treatment was 137/μL (IQR: 58–268), but this value was missing in charts for a quarter (24.3%) of patients (Table 1).

**Table 1:** Demographic and clinical characteristics of HIV-positive TB patients (N = 466).

Variable	N	%
**Gender**		
Male	240	51.5
Female	225	48.3
Missing	1	0.2
**Age**		
15–24	35	7.5
25–49	388	83.3
50+	40	8.6
Missing	3	0.6
**Facility type**		
Clinic	162	34.8
Health Centre	112	24.0
Hospital	192	41.2
**Region**		
Hhohho	146	31.3
Lubombo	65	13.9
Manzini	160	34.3
Shiselweni	95	20.4
**Type of Patient**		
New	393	84.3
Relapse	39	8.4
Return after default	7	1.5
Transfer in	6	1.3
Other	18	3.9
Missing	3	0.6
**Disease Site**		
Pulmonary	391	83.9
Extra-pulmonary	75	16.1
**CD4 at TB Treatment Start**		
<50/μL	79	17.0
50-200/μL	149	32.0
>200/μL	125	26.8
Missing	113	24.2
**GeneXpert Result**		
MTB not detected	124	26.6
MTB detected	284	60.9
Missing	58	12.5
**Missed appointments**		
No	328	70.4
Yes	125	26.8
Missing	13	2.8
**TB Treatment Outcome**		
Cured	241	51.7
Completed	155	33.3
Failure	12	2.6
Default	13	2.8
Died	33	7.1
Transfer Out	11	2.4
Missing	1	0.2
**TOTAL**	**466**	

### Timing of ART uptake for HIV-positive TB patients

Of the 466 HIV-positive TB patients, 189 (40.6%) were on ART prior to starting TB treatment. Two patients (0.4%) were never found to have started ART and one (0.2%) did not have timing of ART documented. Of the 274 other patients who were eligible to begin ART while on TB treatment, only five (1.8%) did not receive it within six months of TB treatment initiation. All five of these patients completed TB treatment or had documented cure, four had a CD4 cell count reported at TB treatment initiation (all between 50 and 200/μL), and only one was reported to have missed appointments.

The median time to ART initiation among the 274 patients overall was 15 days (IQR: 14–28). Although almost 90% started ART within eight weeks of TB treatment initiation, only 25 of the 55 patients (45.5%) with a reported CD4 count less than 50 cells/μL started ART within the recommended two weeks. Conversely, of the 175 patients with a reported CD4 count of 50 cells/μL or above, 155 (88.6%) started ART within the recommended eight weeks. Notably, of the 44 (16.1%) patients with no documented CD4 cell count, 47.7% began ART within two weeks, and 93.2% within eight. The timing of ART initiation stratified by baseline CD4 cell count is presented in Table 2.

**Table 2:** Timing of ART initiation after the start of TB treatment (n = 274).

Baseline CD4	≤ 2 weeks(N/%)	2–8 weeks(N/%)	8 weeks-6 months (N/%)	> 6 months(N/%)
<50/μL (n = 55)	25 (45.5)*	25 (45.5)	5 (9.1)	0 (0.0)
50-200/μL (n = 102)	40 (39.2)*	51 (50.0)*	7 (6.9)	4 (3.9)
>200/mL (n = 73)	26 (35.6)*	38 (52.1)*	9 (12.3)	0 (0.0)
Missing (n = 44)	21 (47.7)*	20 (45.5)	2 (4.5)	1 (2.3)
**Total (n = 274)**	**112 (40.9)**	**134 (48.9)**	**23 (8.4)**	**5 (1.8)**

*Timely ART, defined as within two weeks for patients with a CD4 <50/μL or missing, and as within eight weeks for all others.

Using the thresholds for “timely ART” according to baseline CD4 cell count, we found that 201 of the 274 patients (73.3%; denoted by asterisks in Table 2) received timely ART after TB treatment initiation; this proportion is 72.8% when including the two patients who were never found to have started ART. Missing CD4 cell count was not significantly associated with timely ART initiation compared with a CD4 count less than 50 cells/μL (adjusted odds ratio [AOR] 1.2, 95% CI: 0.4–3.3). However, patients with a CD4 count of 50 to 200 cells/μL or greater than 200/μL had significantly higher odds of receiving timely ART than patients with a CD4 less than 50 cells/μL, with adjusted odds ratios of 11.5 (95% CI: 5.0–26.6) and 9.6 (95% CI: 4.6–19.9), respectively (Table 3).

**Table 3:** Patient characteristics associated with timely* ART for HIV+ TB patients not already on ART at TB treatment initiation (N = 276).

Variable	N (%)	OR (95% CI)	p-value	AOR (95% CI)**	p-value
**Gender**					
Male	111 (73.5)	1.08 (0.67, 1.73)	.7530		
Female	90 (72.0)	ref			
**Age**			.0414		.3578
15–24	17 (81.0)	0.45 (0.07, 2.87)		0.30 (0.07, 2.64)	
25–49	163 (70.6)	0.25(0.05, 1.20)		0.27 (0.04, 1.67)	
50+	19 (90.5)	ref		ref	
**Facility type**			.5986		
Clinic	77 (70.0)	0.83 (0.30, 2.31)			
Health Centre	45 (76.3)	1.14 (0.45, 2.87)			
Hospital	79 (73.8)	ref			
**Region**			.0165		**.0006**
Hhohho	59 (67.1)	0.41 (0.17, 0.97)		0.24 (0.11, 0.53)	
Lubombo	20 (83.3)	1.00 (0.55, 1.81)		0.79 (0.42, 1.49)	
Manzini	77 (70.0)	0.47 (0.22, 0.99)		0.31 (0.09, 1.13)	
Shiselweni	45 (83.3)	ref		ref	
**Type of Patient**			.2519		
New	182 (71.7)	0.45 (0.11, 1.77)			
Other	17 (85.0)	ref			
**Disease Site**			.6093		
Pulmonary	180 (73.2)	1.17 (0.64, 2.13)			
Extra-pulmonary	21 (70.0)	ref			
**CD4, TB Treatment Start**			<.0001		**<.0001**
<50/μL	25 (45.5)	ref		ref	
50-200/μL	91 (89.2)	9.93 (3.75, 26.28)		11.48 (4.96, 26.56)	
>200/μL	64 (86.5)	7.68 (3.66, 16.13)		9.59 (4.63, 19.87)	
Missing	21 (46.7)	1.05 (0.38, 1.81)		1.08 (0.39, 2.97)	
**GeneXpert Result**			.3125		
MTB not detected	46 (68.7)	ref			
MTB detected	138 (75.8)	1.43 (0.71, 2.87)			
**Missed Appointments on TB Treatment**			.7200		
Yes	42 (71.2)	0.89 (0.46, 1.72)			
No	159 (73.6)	ref			
**TB Outcomes**			.2905		
Cured or Completed	170 (71.1)	0.49 (0.13, 1.83)			
Not cured or completed	30 (83.3)	ref			

*Timely ART was defined as within two weeks for patients with a CD4 <50/μL or missing, and as within eight weeks for all others.

**Variables with p<0.20 in a univariate model were included in the multiple regression model to estimate AORs and confidence limits. A p-value of 0.05 was considered statistically significant in the multiple regression model.

In addition, geographic region was found to be significantly associated with timely ART initiation in multivariate analysis, with patients in Hhohho and Manzini having much lower odds of timely ART as compared to those in Shiselweni (AORs 0.2 [95% CI: 0.1–0.5] and 0.3 [95% CI: 0.1–1.1], respectively). Gender, age, facility type, patient type, disease site and missing appointments were not found to be significantly predictive factors.

### TB treatment outcomes

TB cure or treatment completion rate at six months was 85% among all 466 HIV-positive TB patients (Table 1), and 71.1% among the 276 not already on ART when they began TB treatment (Table 3). For this latter group, TB treatment outcomes were not significantly associated with timely ART initiation.

## Discussion

Over the last decade, after declaring HIV and TB to be public health emergencies, the Government of the Kingdom of Swaziland and partners have prioritized efforts to strengthen collaborative TB/HIV activities, and to reduce the burden of both diseases in the country [13–14]. This retrospective study of known HIV-positive TB patients demonstrates the relative success of integrated and co-located TB/HIV services in Swaziland in terms of ART uptake. Approximately 40% of people diagnosed with TB were already on ART before they began TB treatment, and 99% were on ART by six months after TB treatment initiation. This latter statistic demonstrates near-perfect compliance with 2015 national guidelines to provide ART to all known HIV-positive TB patients regardless of CD4 cell count [15], and is exceptional when compared with published literature from other integrated settings in sub-Saharan Africa (which have shown ART uptake among co-infected patients ranging from 58–80%) [16–22]. ART uptake elsewhere among HIV-positive TB patients is even lower in non-integrated settings [23–25], and was estimated by WHO at the time of this study to be only 78% worldwide [1].

In addition to overall ART uptake, the timing of ART initiation relative to the start of TB treatment is important [5,10–12]. In our study population of co-infected patients not already on ART at the time of TB treatment initiation, almost 90% initiated ART within eight weeks as per Swaziland national guidelines, with a median time to initiation of 15 days. This also far surpasses achievements in similar settings, which have found 59–78% ART initiation within eight weeks [16,18–19,21–22] and median time to initiation ranging from 34 to 75 days (except in one Malawi study, which achieved a median 14 days to ART initiation in the context of newly integrated TB/HIV guideline implementation) [16–19,21]. This relative success in Swaziland perhaps validates recent initiatives such as task-shifting to nurse-led ART initiation and successful integration of HIV services into TB clinics. Indeed in Shiselweni, the region supported by Médecins Sans Frontières (MSF) to initially and most rapidly implement task-shifting and treatment decentralization within Swaziland, co-infected patients had higher odds of receiving timely ART uptake in our study than in other regions.

Despite this overall success, when stratified by baseline CD4 cell count, we found that only about three quarters (73%) of co-infected patients not already on ART actually received what is considered to be timely ART per WHO best practice guidelines. Although almost 90% of those with a CD4 cell count over 50/μL began ART within the recommended eight weeks of TB treatment initiation, only about 45% of patients with a CD4 cell count less than 50/μL (those with highest mortality risk who would benefit most from early ART) were found to have received ART within two weeks. Although all patients in this latter group were eventually documented to have started ART (indicating that they remained in care at least until six months of follow up), this finding indicates room for improvement. The fact that the proportion of patients receiving ART within two weeks was only slightly higher for patients with a CD4 cell count less than 50/μL than for those with a CD4 cell count more than 50/μL suggests that providers were not optimally differentiating the timing of ART for co-infected patients based on their CD4 count. In addition to reinforcement of national guidelines, sensitization of providers to the benefits of early ART—*especially* for the sickest TB patients in whom they may currently be hesitating to start ART soon after TB treatment (perhaps due to concerns about pill burden and/or immune reconstitution inflammatory syndrome)—will be critical.

CD4 cell count at TB treatment initiation was not documented in almost a quarter of patients, which is consistent with reports of reagent stock outs and/or other laboratory shortages during the study period. Interestingly, PLHIV with a missing CD4 cell count had similar timing of ART initiation to those with a documented CD4 less than 50 cells/μL. We suggest that, for those in whom a CD4 cell count is missing (e.g., not point-of-care or otherwise easily available), timely ART should mean initiation within two weeks of TB treatment—especially in settings where PLHIV still present late to care—to ensure early ART for potentially severely immunocompromised patients who may be asymptomatic at WHO staging. This may become additionally important if programs opt-out of baseline CD4 testing in the context of new WHO guidance to treat all PLHIV regardless of their immune status [9].

Overall, 85% of co-infected patients in this cohort were documented to have achieved TB cure or to have completed TB treatment, compared with a 71% WHO-estimated TB cure rate for PLHIV in 2014, and a 79% treatment success rate reported in Swaziland’s 2014 Annual TB Programme Report of national-level data [26–27]. Less than 3% of patients experienced documented treatment failure or defaulted from treatment, although 7% died during follow-up. Timely ART initiation relative to TB treatment did not result in significantly different TB treatment outcomes at six months. This has been shown in other studies as well, although several randomized controlled trials have supported the fact that early ART does improve survival specifically for those with very low CD4 cell counts [10–12,24,28–30]. Unfortunately, we were not able to assess HIV treatment outcomes for our study population in order to infer any effect of ART timing on these outcomes.

The major strength of this study is that it drew from routine programmatic data from a diverse set of public health facilities in Swaziland, three years after the national TB/HIV guidelines were revised, and can thus be used for programmatic evaluation and planning. There are, however, some limitations. Sites were selected purposefully and thus may not be nationally representative, and the number of patients included in the study who were not already on ART at the time of TB treatment limited us to detection of medium to large effect sizes (i.e. odds ratios). Using a retrospective chart review, we were unable to make temporal observations (such as potential reasons for ART delay), and data accuracy depended on how well patient-level data was entered into site registers and charts. Additionally, because of logistical constraints we only followed patients for six months after TB treatment initiation, and thus were unable to measure longer-term HIV treatment outcomes (such as viral suppression), which may have been affected by timeliness of ART. Finally, we cannot specifically draw conclusions about improvements specifically due to integration of TB/HIV services in Swaziland, since this is the first time systematic data has been collected in the country on ART timing relative to TB treatment. However, results from this study can serve as a baseline from which to measure future progress.

This assessment of TB/HIV services provided in Swaziland’s co-located clinics found an exceptionally high degree of timely ART uptake among known HIV-positive TB patients, although less so among patients with low or missing CD4 cell counts. This data is being used as an assessment of program quality, and efforts are occurring in country to address delayed ART uptake among patients with CD4 counts less than 50 cells/μL and the high degree of missing CD4 cell counts. However, unlike several studies in other settings, this provides evidence that near-perfect levels of ART uptake for HIV-positive TB patients (albeit with some delay) *can* be achieved in integrated but low-resource TB/HIV settings, advancing efforts to reach ambitious 90-90-90 targets and to reduce HIV-associated TB incidence in Swaziland. Indeed, as ART has been scaled up in Swaziland, TB case notification rates have concurrently declined [13], and a national TB prevalence survey currently underway will hopefully confirm a true decrease in HIV-associated TB. However, further intensification of TB case finding (currently estimated at only 61% by WHO) [2] would likely unearth more “missed” HIV-positive TB cases who could benefit from timely ART services.

## Supporting information

S1 File Minimal dataset. (DTA)
